# The Paradox of Digital Monitoring: A Cross-Sectional Study of mHealth Adoption and Its Association with Psychological Distress Among Pregnant Women in Romania

**DOI:** 10.3390/healthcare14091216

**Published:** 2026-05-01

**Authors:** Roxana Ana Maria Dinescu, Alexandru Catalin Motofelea, Paul-Manuel Luminosu, Alin Stefan Constantin, Ioan Sas

**Affiliations:** 1 Department of Doctoral Studies, “Victor Babeș” University of Medicine and Pharmacy, Eftimie Murgu Square No. 2, 300041 Timisoara, Romania; roxana.dinescu@umft.ro (R.A.M.D.); paul.luminosu@umft.ro (P.-M.L.); 2 Department of Obstetrics and Gynecology, “Victor Babeș” University of Medicine and Pharmacy, 300041 Timisoara, Romania; sas.ioan@umft.ro; 3 Center for Molecular Research in Nephrology and Vascular Disease, Faculty of Medicine, “Victor Babeș” University of Medicine and Pharmacy, 300041 Timisoara, Romania; 4 Clinic of Anaeshtesia and Intensive Care, Emergency County Hospital Pius Brinzeu, 325100 Timisoara, Romania; 5 Doctoral School, Carol Davila University of Medicine and Pharmacy Bucharest, Strada Eroii Sanitari nr 8, 050474 București, Romania

**Keywords:** mHealth, perinatal mental health, pregnancy, anxiety, depression, Romania, digital health paradox, wearable devices

## Abstract

**Background:** Digital health (mHealth) interventions are increasingly integrated into maternity care to improve health literacy and reassure expectant mothers. However, the “double-edged sword” of continuous monitoring may be associated with heightened anxiety. This study aimed to describe mHealth usage patterns and investigate the association between technology engagement and mental health outcomes among pregnant women in Romania, where perinatal distress is a significant public health challenge. **Methods:** This observational, cross-sectional study included 100 pregnant and immediate postpartum women at a tertiary maternity unit in Romania. Participants were stratified into mHealth Users (n = 52) and Non-Users (n = 48). Validated instruments, including the PHQ-9, GAD-7, and EPDS, assessed depressive and anxiety symptoms. Predictors of adoption were identified using multivariable binary logistic regression. **Results:** mHealth users were predominantly from urban environments (80.8% vs. 54.2%, *p* = 0.004) and reported higher rates of daily physical activity (*p* < 0.001). Users experienced significantly higher median scores for depression (PHQ-9: 6 vs. 4, *p* = 0.047), generalized anxiety (GAD-7: 7 vs. 6, *p* = 0.015), and pregnancy-specific anxiety (35 vs. 29.5, *p* = 0.028) compared to non-users. In the multivariable model, high psychological distress (OR 0.08 for low-stress vs. high-stress, *p* = 0.009) and urban residency (*p* = 0.043) were independent predictors of mHealth adoption. Notably, 96.2% of users shared their digital data with healthcare providers. **Conclusions:** mHealth adoption in this population is characterized by a “paradox of monitoring,” where usage is strongly associated with pre-existing psychological vulnerability and associated with higher distress. While these tools serve as markers for mental health risk, the high rate of data sharing offers a clinical opportunity for a hybrid model of care. Obstetricians should view high digital engagement as a prompt for targeted mental health screening and proactively mediate patient-generated data to mitigate anxiety.

## 1. Introduction

Pregnancy and the transition to motherhood represent a major life phase characterized by profound physiological changes and role adjustments. While often a period of anticipation, it is also a time of increased uncertainty where women must adapt to new responsibilities regarding their health and that of their unborn child [1]. In Romania, perinatal mental health constitutes a significant public health challenge. Recent epidemiological data indicates a high burden of distress, with postpartum depression (PPD) prevalence estimates ranging from 18.8% during the COVID-19 pandemic [2] to as high as 26.1% in the immediate postpartum period [3]. More recent findings from Western Romania utilizing the Edinburgh Postnatal Depression Scale (EPDS) suggest that when screening for broader depressive symptoms (EPDS ≥ 10), nearly one-third (32.8%) of women may be affected [4].

To navigate these uncertainties and improve health literacy, global health strategies have increasingly integrated digital interventions into maternity care [1]. Consequently, the adoption of mobile health (mHealth) has become widespread; research during the pandemic era indicated that 77.9% of pregnant women utilized mobile applications to track pregnancy changes and access health information [5]. These technologies are often valued by women for their flexibility, the ability to provide personalized follow-up, and the potential to reduce travel time to clinical appointments [6].

However, the relationship between mHealth engagement and maternal mental well-being is complex. While these tools are designed to provide reassurance, theoretical frameworks describe smart health monitoring technologies (SHMTs) as a “double-edged sword” [7]. Tretter (2024) argues that while continuous monitoring can mitigate certain uncertainties, it simultaneously generates new anxieties regarding data interpretation and the reliability of measurements, potentially leading to unnecessary stress [7]. This “paradox of monitoring” is supported by qualitative evidence suggesting that online information seeking can lead women down a “rabbit hole” of worst-case scenarios, resulting in heightened worry despite an initial intent to find reassurance [8].

Furthermore, empirical evidence suggests that high technology adoption may be a marker of, rather than a cure for, underlying distress. Research by Özkan Şat and Yaman Sözbir (2021) identified that problematic health situations and experiencing complications during pregnancy were significant predictors of mobile application usage [5]. Similarly, qualitative studies have noted that the large amount of data generated by remote monitoring can increase stress among pregnant women [6]. These findings support the hypothesis that health anxiety may drive technology adoption, as women with higher baseline concerns seek self-monitoring tools for reassurance in the face of perceived risks.

Despite the high prevalence of perinatal depression in Romania [2,3] and the growing integration of digital tools in prenatal care, there is a paucity of research examining the association between mHealth adoption and mental health outcomes in this specific population. This study aims to describe the patterns of mHealth use among pregnant women in Romania and investigate the association between technology engagement, anxiety, depression, and perceived health status.

## 2. Materials and Methods

### 2.1. Study Design and Setting

This observational, cross-sectional study was conducted between February 2025 and February 2026 at Spitalul Clinic de Obstetrică-Ginecologie “Prof. Dr. Panait Sîrbu”, a tertiary maternity unit serving Bucharest, Romania. The study was designed to evaluate the prevalence of mobile health (mHealth) utilization among pregnant and immediate postpartum women and to assess its relationship with mental health outcomes and perceived health status. The reporting of this study conforms to the Strengthening the Reporting of Observational Studies in Epidemiology (STROBE) guidelines [9].

### 2.2. Participants and Eligibility Criteria

The study population consisted of pregnant women admitted for prenatal care or delivery and women in the immediate postpartum period (days 2–4). Participants were recruited using a convenience sampling method. The inclusion criteria were: (1) age ≥ 18 years; (2) ability to read and understand the Romanian language; and (3) willingness to provide informed consent. Exclusion criteria included a history of psychotic disorders, current substance abuse, or severe obstetric emergencies that precluded the ability to complete the questionnaire.

A total of 100 women were included in the final analysis. Participants were stratified into two primary groups based on their self-reported technology usage: mHealth Users (n = 52), defined as women who used mobile applications or wearable devices for pregnancy monitoring at least occasionally, and Non-Users (n = 48), defined as women who relied solely on standard medical care without digital supplementation.

### 2.3. Data Collection and Instruments

Data were collected using a structured self-administered questionnaire divided into three sections: sociodemographic/clinical characteristics, mHealth usage patterns, and standardized psychological assessments. The investigator-developed questionnaire was constructed based on a review of contemporary digital health literature and clinical expertise. Prior to formal data collection, the questionnaire was reviewed by an expert panel of two obstetricians and a clinical psychologist for face validity, and pilot-tested on a small group of patients (n = 10) to ensure the readability and clarity of the questions.

### 2.4. Sociodemographic and Clinical Data

We collected data regarding maternal age, environment of residence (urban/rural), education level, parity, gravidity, gestational age, history of mental health disorders, and obstetric comorbidities (e.g., hypertension, gestational diabetes).

### 2.5. mHealth Utilization Profile

For the purposes of this study, ‘mHealth applications’ were broadly defined to include digital tools utilized during the perinatal period, such as week-by-week fetal development trackers, fetal movement (kick count) monitors, maternal symptom trackers, contraction timers, and early postpartum or breastfeeding support applications. A specific investigator-developed questionnaire was used to assess digital behavior. This section evaluated the type of technology used (mobile applications vs. wearables), frequency of use (daily vs. occasional), specific health parameters monitored (e.g., fetal activity, blood pressure), and data-sharing behaviors with healthcare providers. Participants also rated perceived benefits (e.g., anxiety reduction) and barriers (e.g., cost, technical issues).

### 2.6. Psychological Measures

Mental health outcomes were assessed using internationally validated instruments. For the PHQ-9, GAD-7, and EPDS, we utilized the official Romanian versions. For the SF-36, where a validated Romanian version was not available for this specific population, a standard forward–backward translation procedure was employed by bilingual medical professionals to ensure conceptual equivalence.

### 2.7. Depressive Symptoms (PHQ-9)

Depressive symptoms were assessed using the PHQ-9, a 9-item instrument based on DSM-IV criteria. Participants rated the frequency of symptoms over the last two weeks on a scale from 0 (“not at all”) to 3 (“nearly every day”). The total score ranges from 0 to 27. We utilized the official Romanian translation provided by the MAPI Research Institute [10,11].

### 2.8. Generalized Anxiety (GAD-7)

Anxiety symptoms were measured using the GAD-7, a 7-item scale designed to screen for generalized anxiety disorder. Items are scored from 0 to 3, yielding a total score between 0 and 21. We utilized the official Romanian translation provided by the MAPI Research Institute [12,13].

### 2.9. Postnatal Depression (EPDS)

The EPDS is a 10-item self-report scale specifically designed to screen for depression in the perinatal period. Each item is scored on a 4-point scale (0–3), with a maximum total score of 30. This study used the Romanian version validated by Tigan et al., which has demonstrated satisfactory psychometric properties in the Romanian population [14,15].

### 2.10. Pregnancy-Specific Anxiety

Pregnancy-specific anxiety was evaluated using the 33-item Pregnancy-Specific Anxiety Tool (PSAT), which assesses six dimensions: baby’s health, labor/well-being, postpartum, career/finance, support, and severity. A score higher than 10 was used as the threshold to identify women requiring further clinical assessment. Since a validated Romanian version was not previously available, the instrument underwent a professional forward–backward translation pathway conducted by bilingual medical experts to ensure conceptual and linguistic equivalence [16].

### 2.11. Perceived Health (SF-36)

Perceived health status was evaluated using the SF-36. This 36-item survey measures eight domains of health: physical functioning, role limitations due to physical health, role limitations due to emotional problems, energy/fatigue, emotional well-being, social functioning, pain, and general health. For this study, we focused specifically on the General Health and Mental Health subscales [17].

### 2.12. Instrument Reliability

To confirm the internal consistency of the psychometric instruments within our specific Romanian cohort, Cronbach’s alpha coefficients were calculated for all scales. All instruments demonstrated high reliability, exceeding the standard threshold of 0.70. The calculated alpha values were: Depressive Symptoms (PHQ-9) α = 0.891; Generalized Anxiety (GAD-7) α = 0.934; Postnatal Depression (EPDS) α = 0.872; Pregnancy-Specific Anxiety α = 0.823; and Perceived Health (SF-36) α = 0.881.

### 2.13. Sample Size Estimation

The minimum sample size required for this study was calculated using the OpenEpi (version 3.01; Open Source Epidemiologic Statistics for Public Health, Atlanta, GA, USA) online sample size calculator was used (available at: https://www.openepi.com/SampleSize/SSPropor.htm, accessed on 15 March 2026). Based on a standard 95% confidence level, a conservative response distribution of 50%, and a margin of error set at 10%, the power analysis indicated that a minimum of 97 participants were needed. Our final sample of 100 participants satisfied this requirement. While a 5% margin of error is standard, a 10% margin was deemed acceptable for this exploratory, single-center study due to resource and time constraints during the study period. Furthermore, our sample of 100 participants provides sufficient statistical power for the multivariable logistic regression, satisfying the ‘rule of 10’ events per predictor variable.

#### Ethical Considerations

The study was conducted in accordance with the Declaration of Helsinki. Ethical approval was obtained from the Ethics Council of Spitalul Clinic de Obstetrică-Ginecologie “Prof. Dr. Panait Sîrbu”, Bucharest, Romania (Protocol No. 1/25.02.2025). All participants provided written informed consent prior to data collection, and all data were anonymized to ensure confidentiality. To ensure data protection and confidentiality, all participant data were pseudonymized upon collection. Digital records were stored on a password-protected, encrypted local server accessible only to the primary investigators, in strict compliance with General Data Protection Regulation (GDPR) guidelines.

### 2.14. Statistical Analysis

Data analysis was performed using IBM SPSS version 26.0. Descriptive statistics were used to characterize the study population, with categorical variables presented as frequencies (n) and percentages (%). Continuous variables were assessed for normality using the Shapiro–Wilk test. As the outcome measures—including PHQ-9, GAD-7, EPDS, and SF-36 scores—demonstrated non-normal distributions, these data are reported as Medians and Interquartile Ranges (IQRs).

Bivariate comparisons between mHealth users and non-users for demographic and clinical characteristics were conducted using the Pearson Chi-square test or Fisher’s exact test for categorical data. To compare mental health and quality of life outcomes between users and non-users, as well as between daily and occasional users, the Mann–Whitney U test was employed.

To identify independent predictors of mHealth adoption, a multivariable binary logistic regression model was constructed. Predictors were selected based on clinical relevance and a bivariate significance threshold of *p* < 0.25 (Hosmer–Lemeshow criterion). The model included environment, psychological support, physical activity, mental health history, stress levels, and prenatal consultation frequency. Model fit was evaluated using the Omnibus Tests of Model Coefficients and the Hosmer–Lemeshow goodness-of-fit test. Prior to running the final model, potential multicollinearity among the independent variables was assessed using the Variance Inflation Factor (VIF). All included variables demonstrated a VIF well below the acceptable threshold of 5 (or 2.5), indicating no significant multicollinearity. Results are reported as Odds Ratios (ORs) with corresponding 95% Confidence Intervals (CIs). For all analyses, a two-tailed *p*-value of < 0.05 was considered statistically significant.

## 3. Results

The demographic, obstetric, and clinical characteristics of the study population (n= 100) stratified by mHealth usage are presented in Table 1 and Supplementary Table S1. Significant geographical and lifestyle disparities were observed, with mHealth users being predominantly from urban environments (80.8% vs. 54.2%, *p* = 0.004) and demonstrating higher levels of physical activity (28.8% daily vs. 4.2%, *p* < 0.001) compared to non-users. Clinically, mHealth users reported a higher prevalence of psychological and obstetric risk factors, including a history of depression (51.9% vs. 27.1%, *p* = 0.036), a history of anxiety or mental disorders (65.4% vs. 39.6%, *p* = 0.010), and higher rates of hypertension (26.9% vs. 10.4%, *p* = 0.034).

Furthermore, users experienced significantly poorer sleep quality (75% reported disorders vs. 52.1%, *p* = 0.033) and higher levels of psychological distress, with 46.2% reporting high stress compared to 22.9% of non-users (*p* = 0.018). Engagement with healthcare was also notably higher among users, who attended regular prenatal consultations more frequently (82.7% vs. 64.6%, *p* = 0.024) and were more likely to utilize formal psychological support (40.4% vs. 8.3%, *p* < 0.001). These findings suggest that mHealth adoption in this Romanian sample is driven by a combination of urban residency, health-conscious behaviors, and a greater clinical or psychological need for monitoring.

Among the 52 mHealth users, wearable devices (smartwatches/bands) were slightly more prevalent than mobile applications (51.9% vs. 48.1%), with physical activity (53.8%) and blood pressure (19.2%) being the primary parameters monitored. Usage was largely occasional (57.7%), yet 96.2% of users shared their data with physicians at least occasionally. The leading perceived benefit was the capacity for continuous monitoring (59.6%), followed by anxiety reduction (32.7%), while limited technology access (59.6%) and high costs (25%) remained the most significant barriers to use. Despite these challenges, 71.2% found the technology easy to integrate into daily life, and 100% perceived at least a partial improvement in maternal–fetal outcomes, with all users indicating they would recommend mHealth tools to others (Table 2).

The comparison of validated mental health instruments revealed that mHealth users experienced significantly higher levels of psychological distress compared to non-users. Specifically, users demonstrated significantly higher median scores for depression (PHQ-9:6 vs. 4, *p* = 0.047), generalized anxiety (GAD-7:7 vs. 6, *p* = 0.015), and pregnancy-specific anxiety (35 vs. 29.5, *p* = 0.028). While median scores for postnatal depression (EPDS) were also higher in the user group (15 vs. 11.5), this difference did not reach statistical significance (*p* = 0.085). Similarly, no significant disparities were observed between the groups regarding perceived general health (*p* = 0.154) or mental health subscales (*p* = 0.942) of the SF-36 (Table 3).

A multivariable binary logistic regression was performed to identify independent predictors of mHealth technology adoption among the study participants. After adjusting for clinical and demographic confounders, three factors emerged as significant independent predictors of mHealth use: environment, current stress level, and physical activity (Table 4).

Participants residing in rural environments had 70% lower odds of utilizing mHealth tools compared to those in urban areas (OR 0.30, 95% CI 0.10–0.96, *p* = 0.043). Notably, high psychological distress was a primary driver of technology engagement; women reporting low stress levels were significantly less likely to adopt mHealth technologies compared to those experiencing high stress (OR 0.08, 95% CI 0.01–0.52, *p* = 0.009). Furthermore, a sedentary lifestyle was inversely associated with mHealth use, as participants with no physical activity (OR 0.05, 95% CI 0.01–0.56, *p* = 0.015) or only occasional activity (OR 0.13, 95% CI 0.02–0.86, *p* = 0.035) demonstrated significantly lower odds of adoption compared to those exercising daily. Interestingly, mental health history, prenatal consultation frequency, and psychological support did not remain significant predictors in the multivariable model, suggesting their influence may be mediated by stress levels and environmental factors.

A subgroup analysis was conducted among mHealth users (n = 52) to determine if the frequency of technology engagement (daily vs. occasional) was associated with differing mental health profiles or data-sharing behaviors. Despite the variation in usage frequency, no statistically significant differences were observed between daily (n = 22) and occasional users (n = 30) across all primary mental health outcomes (Table 5).

## 4. Discussion

This study provides the first comprehensive assessment of mobile health (mHealth) adoption among pregnant women in a Romanian tertiary center, revealing a complex relationship between digital engagement and maternal mental health. Contrary to the marketing narrative that mHealth tools empower and reassure users, our findings indicate a distinct “paradox of monitoring.” Women who utilized mHealth tools demonstrated significantly higher scores for depression (PHQ-9), generalized anxiety (GAD-7), and pregnancy-specific anxiety compared to non-users. Furthermore, our data highlights a persistent digital divide, where adoption is driven not merely by medical necessity, but by urban residency and pre-existing psychological distress.

A central finding of this study is the significant association between mHealth usage and elevated psychological distress. While it is tempting to suggest that technology induces anxiety (“technostress”), our regression analysis identifies high baseline stress as a powerful independent predictor of adoption (OR 0.08 for low-stress vs. high-stress women). This supports the “health anxiety” hypothesis: women with elevated baseline anxiety or a history of mental health disorders—who comprised 65.4% of our user group—likely self-select into technology use as a mechanism of “reassurance seeking” [18].

This interpretation aligns with the theoretical framework of “cyberchondria,” defined not as a disease, but as a behavioral pattern where distress drives online searching, yet the ambiguity of the information found fails to provide lasting relief, leading to a cycle of repeated monitoring [1,2]. In our sample, although “continuous monitoring” was the primary perceived benefit (59.6%), the elevated GAD-7 scores suggest that this monitoring did not translate into clinical reassurance. This reflects the “reassurance-seeking loop” described in cognitive–behavioral models of health anxiety, where the relief obtained from checking symptoms is short-lived, necessitating further digital engagement to manage intolerance of uncertainty [19,20].

The inability of these tools to lower anxiety scores may also stem from the content and functionality of the apps themselves. A recent systematic analysis of 35 popular pregnancy apps identified a phenomenon of “feature creep,” where developers include non-evidence-based tracking tools—such as fetal kick counters and heart rate monitors—that promote hyper-vigilance and unjustified anxiety [21]. Furthermore, while our participants sought reassurance, global data suggests they likely encountered the opposite; a 2024 study of 427 pregnant women found that 35.5% encountered app information they felt was unsafe or conflicted with medical advice [22]. When vulnerable women (such as the 51.9% in our sample with a history of depression) encounter such conflicting data, the app transforms from a tool of support into a source of cognitive dissonance and distress.

Our study identified a profound demographic disparity, with rural women having 70% lower odds of adopting mHealth tools compared to their urban counterparts. This finding must be interpreted within the specific context of Romania’s digital infrastructure. As noted by Buțu et al. (2024), while internet connectivity in rural Romania has improved dramatically from 41.08% in 2014 to 88.12% in 2023, significant qualitative gaps remain regarding digital literacy and network stability [23]. Our results confirm the “Digital Inverse Care Law,” which posits that the populations with the highest health needs often have the least access to digital interventions [24]. In our cohort, the barrier was not a lack of interest, but “Limited Access” (59.6%). As evidenced by recent structural equation modeling in similar populations, “Facilitating Conditions” (access to devices and reliable data) are often the strongest predictor of actual usage behavior, outweighing social influence [25].

Despite the associated anxiety, a highly positive finding was that 96.2% of users shared their digital data with physicians. This challenges the notion that mHealth alienates patients from providers. Instead, it suggests a “hybrid” model of care where women use apps to gather data but rely on clinicians to validate it. This aligns with Australian cohorts where 96.1% of women rated medical practitioners as the most trustworthy source of information, and 62.8% stated they would trust an app more if explicitly recommended by their doctor [22]. Consequently, the high anxiety observed in our users may represent an unaddressed need for clinical interpretation of the data they are collecting.

Our findings stand in contrast to a recent study by Nurdilan and Simge (2026), which reported that pregnancy app users had significantly lower prenatal distress and higher maternal attachment [26]. This discrepancy is likely attributable to inclusion criteria; while Nurdilan excluded women with diagnosed psychiatric illnesses, our study included them, and they formed the majority of our user base. This comparison offers a critical clinical insight: mHealth apps may function as effective support tools for “healthy” pregnant women, but may inadvertently exacerbate distress in women with a history of anxiety or depression by feeding into compulsive monitoring behaviors [26,27].

Several limitations must be acknowledged. First, the cross-sectional design precludes causal inference; we cannot definitively state whether anxiety drives app use or if app use increases anxiety, though the literature supports a bidirectional relationship. Second, the use of convenience sampling from a single tertiary care center introduces potential selection bias. Tertiary centers typically manage a higher volume of complex or high-risk pregnancies, meaning our sample may exhibit higher baseline levels of psychological distress and different health-seeking behaviors compared to women in primary care or rural settings. Consequently, external validity may be limited. Third, we did not assess the specific content of the apps used, preventing us from distinguishing between evidence-based medical apps and general wellness trackers. Fourth, combining pregnant women and immediate postpartum women (days 2–4) in a single analytical sample presents a limitation. While the postpartum participants were reflecting on their mHealth usage during their recent pregnancy, the profound hormonal and psychological shifts occurring immediately after delivery could independently influence their EPDS and PHQ-9 scores, potentially introducing confounding variance. Fifth, self-reported usage frequency may be subject to recall bias. Sixth, the relatively small sample size (N = 100) and the 10% margin of error may limit the precision of our estimates, as reflected by the wide confidence intervals in the regression analysis. Finally, while our study accounted for general medical/family support and basic comorbidities (e.g., hypertension, gestational diabetes), we did not explicitly measure specific partnership status or comprehensive high-risk obstetric diagnoses (e.g., history of pregnancy loss, fetal growth restriction). It is highly plausible that an underlying high-risk pregnancy, rather than the digital application itself, acts as an unmeasured confounder that simultaneously triggers both heightened anxiety and the compensatory use of monitoring apps.

Based on these findings, we recommend that obstetricians view high engagement with pregnancy tracking tools not merely as health consciousness, but as a potential digital marker for underlying psychological distress, prompting targeted mental health screening for these “highly monitored” patients. Clinicians should proactively integrate the review of patient-generated data into routine consultations to correct misinformation and validate findings, thereby transforming the app from a source of anxiety into a bridge for clinical communication [8]. Furthermore, public health strategies in Romania must prioritize digital literacy and infrastructure in rural areas to prevent the widening of the digital inverse care law. Crucially, to move beyond the associations observed in this cross-sectional design and definitively disentangle the “chicken and egg” relationship between anxiety and technology use, future research must prioritize longitudinal and interventional studies such as randomized controlled trials comparing guided versus unguided app use to determine causality.

## 5. Conclusions

In conclusion, mHealth adoption among pregnant women in this Romanian cohort is characterized by a “paradox of monitoring,” where digital engagement is strongly associated with urban residency and elevated levels of stress and anxiety. Given the cross-sectional design of this study, this relationship must be interpreted as associative rather than causal. It is highly plausible that underlying psychological vulnerability, health anxiety, or complex obstetric factors prompt the initial use of these tools, potentially creating a bidirectional cycle of continuous monitoring and distress. While heavy mHealth use may serve as a potential clinical indicator of underlying worry, it also presents a valuable opportunity for a hybrid model of care. The high prevalence of data sharing with healthcare providers suggests that women are actively seeking professional validation of their digital metrics. Therefore, integrating digital tools into prenatal care requires a shift toward active clinical mediation. By collaboratively reviewing and interpreting patient-generated data during consultations, obstetricians can ensure that the search for reassurance does not inadvertently sustain a cycle of anxiety for vulnerable expectant mothers.

## Figures and Tables

**Table 1:** Demographic, obstetric, and clinical characteristics of the study sample (N = 100), stratified by mHealth usage.

Variable	Non-Users (n = 48)	mHealth Users (n = 52)	*p*-Value
Maternal Age			0.130
<25 years	18 (37.5)	9 (17.3)	
25–34 years	13 (27.1)	22 (42.3)	
35–39 years	12 (25)	14 (26.9)	
≥40 years	5 (10.4)	7 (13.5)	
Environment			0.004
Urban	26 (54.2)	42 (80.8)	
Rural	22 (45.8)	10 (19.2)	
Gravidity			0.118
1	26 (54.2)	36 (69.2)	
2	15 (31.3)	14 (26.9)	
≥3	7 (14.6)	2 (3.8)	
Parity			0.209
1 (Primiparous)	28 (58.3)	38 (73.1)	
2	13 (27.1)	11 (21.2)	
≥3	7 (14.6)	3 (5.8)	
History of Depression			0.036
No	35 (72.9)	25 (48.1)	
Yes (During/Before)	13 (27.1)	27 (51.9)	
History Anxiety/Mental Disorders			0.010
No	29 (60.4)	18 (34.6)	
Yes	19 (39.6)	34 (65.4)	
Comorbidities			0.034
None	43 (89.6)	36 (69.2)	
Hypertension	5 (10.4)	14 (26.9)	
Diabetes	0 (0)	2 (3.8)	
Current Stress Level			0.018
Low	11 (22.9)	4 (7.7)	
Moderate	26 (54.2)	24 (46.2)	
High	11 (22.9)	24 (46.2)	
Sleep Quality (Disorders)			0.033
No (Sleep Well)	23 (47.9)	13 (25)	
Yes (Frequent awakening/Insomnia)	25 (52.1)	39 (75)	
Psychological Support (Counseling)			<0.001
No	44 (91.7)	31 (59.6)	
Yes	4 (8.3)	21 (40.4)	
Medical & Family Support			0.031
Yes	25 (52.1)	40 (76.9)	
Partial	20 (41.7)	11 (21.2)	
No	3 (6.3)	1 (1.9)	
Prenatal Consultations			0.024
Regular	31 (64.6)	43 (82.7)	
Occasional	15 (31.3)	5 (9.6)	
None	2 (4.2)	4 (7.7)	
Physical Activity			<0.001
Daily	2 (4.2)	15 (28.8)	
Occasional	33 (68.8)	34 (65.4)	
None	13 (27.1)	3 (5.8)	

Data presented as n (%). *p*-values calculated using Pearson Chi-square test or Fisher’s Exact Test where appropriate. Abbreviations: mHealth, mobile health.

**Table 2:** Patterns of mobile health technology usage, perceived benefits, and barriers among users (n = 52).

Variable	N (%)
Frequency of Use	
Daily	22 (42.3)
Occasional	30 (57.7)
Type of Technology Used	
Mobile Applications	25 (48.1)
Wearable Devices (Smartwatch/Band)	27 (51.9)
Health Parameters Monitored	
Physical Activity	28 (53.8)
Heart Rate	8 (15.4)
Blood Pressure	10 (19.2)
SpO2	4 (7.7)
Other	2 (3.8)
Perceived Benefits	
Continuous Monitoring	31 (59.6)
Anxiety Reduction	17 (32.7)
Early Detection of Problems	2 (3.8)
None/Other	2 (3.8)
Perceived Limitations (Barriers)	
Limited Access to Technology	31 (59.6)
Data Inaccuracy	8 (15.4)
High Costs	13 (25)
Data Sharing with Doctor	
Regularly	17 (32.7)
Occasionally	33 (63.5)
Never/No	2 (3.8)
Perceived Impact on Outcomes	
Significant Improvement	26 (50)
Partial Improvement	26 (50)
No Improvement	0 (0)
Ease of Integration	
Easy	37 (71.2)
Moderate	15 (28.8)
Difficult	0 (0)
Would Recommend to Others	
Yes	52 (100)
No	0 (0)

Data presented as frequency (n) and percentage (%). Abbreviation: SpO2, Oxygen Saturation.

**Table 3:** Comparison of mental health and quality of life scores between mHealth users and non-users.

Outcome Variable	Non-Users (n = 48) Median (IQR)	mHealth Users (n = 52) Median (IQR)	*p*-Value
Depression (PHQ-9)	4 (2–8)	6 (4.25–8)	0.047
Anxiety (GAD-7)	6 (1–7)	7 (6.25–7.75)	0.015
Postnatal Depression (EPDS)	11.5 (6–16)	15 (11.5–15)	0.085
Pregnancy-Specific Anxiety	29.5 (21.5–35.75)	35 (29–39.75)	0.028
SF-36 General Health	67.2 (55.55–72.20)	62 (52.20–72.20)	0.154
SF-36 Mental Health	60 (54–60)	60 (56–68)	0.942

Data presented as Median (IQR) for continuous variables. Mann–Whitney U test used for these non-normally distributed continuous data. Abbreviations: PHQ-9, Patient Health Questionnaire-9; GAD-7, Generalized Anxiety Disorder-7; EPDS, Edinburgh Postnatal Depression Scale; SF-36, Short Form-36 Health Survey.

**Table 4:** Independent Predictors of mHealth Technology Use (n = 100).

Predictor	Category	Odds Ratio (95% CI)	*p*-Value
Environment	Rural (vs. Urban)	0.30 (0.10–0.96)	0.043
Current Stress Level	Low (vs. High)	0.08 (0.01–0.52)	0.009
	Moderate (vs. High)	0.46 (0.13–1.66)	0.236
Physical Activity	None (vs. Daily)	0.05 (0.01–0.56)	0.015
	Occasional (vs. Daily)	0.13 (0.02–0.86)	0.035
Mental Health History	Anxiety (vs. None/Other)	0.92 (0.29–2.91)	0.891
Psychological Support	Partial (vs. Adequate)	0.48 (0.12–1.96)	0.304
Prenatal Consultations	None (vs. Regular)	0.75 (0.10–5.75)	0.780
	Occasional (vs. Regular)	0.32 (0.08–1.26)	0.103

Model Fit: Omnibus Tests of Model Coefficients: *p* < 0.001; Hosmer and Lemeshow Test: *p* = 0.287. Abbreviations: CI, confidence interval; OR, Odds Ratio.

**Table 5:** Comparison of Mental Health Outcomes and Technology Engagement by Frequency of Use (n = 52).

Variable	Occasional Users (n = 30) Median (IQR)	Daily Users (n = 22) Median (IQR)	*p*-Value
Depression (PHQ-9)	6 (4–8)	6 (5–8)	0.723
Anxiety (GAD-7)	7 (5–7)	7 (7–8)	0.500
Postnatal Depression (EPDS)	15 (8–15)	15 (15–15)	0.219
Pregnancy-Specific Anxiety	34.5 (34.5–40)	35 (30–38)	0.704
SF-36 General Health	64.5 (52.2–76.2)	59.7 (52.2–67.2)	0.399
SF-36 Mental Health	60 (56–72)	62 (56–64)	0.708

Continuous variables are presented as Median and Interquartile Range (IQR). Comparisons between daily and occasional users were performed using the Mann–Whitney U test. Abbreviations: PHQ-9, Patient Health Questionnaire-9; GAD-7, Generalized Anxiety Disorder-7; EPDS, Edinburgh Postnatal Depression Scale; SF-36, Short Form-36 Health Survey.

## Data Availability

The data presented in this study are available upon request from the corresponding author. The data are not publicly available due to privacy and ethical restrictions related to the protection of participant confidentiality.

## Supplementary Materials

The following supporting information can be downloaded at: https://www.mdpi.com/article/10.3390/healthcare14091216/s1, Table S1: Clinical characteristics of the study sample (N = 100), stratified by mHealth usage.
