# Supplementary material for: The Paradox of Digital Monitoring: A Cross-Sectional Study of mHealth Adoption and Its Association with Psychological Distress Among Pregnant Women in Romania
Source: Healthcare (Basel). 2026 May 1;14(9):1216. doi: 10.3390/healthcare14091216 (PMC13163444; doi:10.3390/healthcare14091216)
Supplement: Supplementary file 1 [file healthcare-14-01216-s001.zip › healthcare-4231080-supplementary.pdf]

Table S1 Clinical characteristics of the study sample (N = 100), stratified by mHealth usage.

**Variable**	**Non-Users (n=48)**	**mHealth Users (n=52)**	***p*-value**
**Pre-pregnancy Weight**			0.322
< 50 kg	8 (16.7)	6 (11.5)	
50–70 kg	29 (60.4)	29 (55.8)	
71–90 kg	11 (22.9)	14 (26.9)	
> 90 kg	0 (0)	3 (5.8)	
**Weight Gained**			0.827
< 5 kg	2 (4.2)	2 (3.8)	
5–10 kg	26 (54.2)	32 (61.5)	
11–15 kg	17 (35.4)	14 (26.9)	
> 15 kg	3 (6.3)	4 (7.7)	
**Sleep Duration**			0.671
< 6 hours	10 (20.8)	8 (15.4)	
6–8 hours	32 (66.7)	35 (67.3)	
> 8 hours	6 (12.6)	9 (17.3)	
**Alcohol use**			0.511
No	46 (95.8)	51 (98.1)	
Yes	2 (4.2)	1 (1.9)	
**Smoking use**			0.130
No	37 (77.1)	46 (88.5)	
Yes	11 (22.9)	6 (11.5)	

Data presented as n (%). p-values calculated using Pearson Chi-square test or Fisher's Exact Test where appropriate. Abbreviations: kg, kilograms.
